# A Novel Broad-Spectrum Elastase-Like Serine Protease From the Predatory Bacterium *Bdellovibrio bacteriovorus* Facilitates Elucidation of Site-Specific IgA Glycosylation Pattern

**DOI:** 10.3389/fmicb.2019.00971

**Published:** 2019-05-03

**Authors:** Eleni Bratanis, Rolf Lood

**Affiliations:** Division of Infection Medicine, Department of Clinical Sciences Lund, Lund University, Lund, Sweden

**Keywords:** serine protease, Bdellovibrio bacteriovorus, immunoglobilins, glycan analysis, IgA

## Abstract

The increased interest in predatory bacteria due to their ability to kill antibiotic resistant bacteria has also highlighted their inherent plethora of hydrolytic enzymes, and their potential as natural sources of novel therapeutic agents and biotechnological tools. Here, we have identified and characterized a novel protease from the predatory bacterium *Bdellovibrio bacteriovorus*: BspE (Bdellovibrio elastase-like serine protease). Mapping preferential sites of proteolytic activity showed a single proteolytic cleavage site of native plasma IgA (pIgA) in the Fc-tail; as well as in the secretory component (SC) of secretory IgA (SIgA). Proteolysis of other native immunoglobulins and plasma proteins was either absent (IgG1 and 2, IgM, albumin and orosomucoid) or unspecific with multiple cleavage sites (IgG3 and 4, IgE, IgD). BspE displayed a broad activity against most amino acid bonds in shorter peptides and denatured proteins, with a slight preference for hydrolysis C-terminal of Y, V, F, S, L, R, P, E, and K. BspE autoproteolysis results in numerous cleavage products sustaining activity for more than 6 h. The enzymatic activity remained stable at pH 5.0–9.0 but was drastically reduced in the presence of MnCl_2_ and completely inhibited by ZnCl_2_. The hydrolysis of pIgA was subsequently utilized for the specific glycan characterization of the released pIgA Fc-tail (Asn^459^). Besides contributing to the basic knowledge of *Bdellovibrio* biology and proteases, we propose that BspE could be used as a potential tool to investigate the importance, and biological function of the pIgA Fc-tail.

IMPORTANCE

Antibodies are well-established as key components of the immune system, and the importance of antibody glycosylation is steadily gaining recognition. Modifications of antibodies by glycosylation creates a vast repertoire of antibody glycovariants with distinctive and diverse functions in the immune system. Most of the available information regarding antibody glycosylation is based on studies with IgG, which have contributed greatly to the advance of therapeutic antibody treatments. However, much is still unknown regarding the importance of glycosylation and the Fc-structure for the remaining antibody classes. Such research has proven to be technically challenging and demonstrates a need for novel tools to facilitate such investigations. Here we have identified and characterized a novel protease from *B. bacteriovorus*, facilitating the study of plasma IgA by cleaving the Fc-tail, including the Asn^459^ N-glycan. This further highlights the potential of *B. bacteriovorus* as a source to identify potential novel biotechnological tools.

## Introduction

With the advent of an increasing number of multi-resistant bacteria, researchers have started looking into alternatives to antibiotics. One of these alternatives is predatory bacteria (Shatzkes et al., 2015; Gupta et al., 2016; Negus et al., 2017). One of the most commonly studied predatory bacteria is *Bdellovibrio bacteriovorus*, commonly found in soil and water environments, as well as in the intestinal tract of mammalians (Rendulic et al., 2004; Sockett and Lambert, 2004). *Bdellovibrio* has a biphasic lifestyle with a motile phase during which it searches for and attaches to the outer membrane of the Gram negative prey bacteria, and an intraperiplasmic parasitizing growth phase, where upon invasion it exploits the prey for nutrients, ending with exhaustion and killing of the prey (Rendulic et al., 2004; Lambert et al., 2010; Baker et al., 2017). Although the underlying mechanisms are not fully understood, it is well-known that this small predator has the ability to switch from a host-dependent (HD) to a host-independent (HI) lifestyle when grown in nutrient rich media (Cotter and Thomashow, 1992; Sockett, 2009), thus allowing it to survive even in prey-free environments as long as the nutrient levels are sufficient. *Bdellovibrio* has a broad host range, although restricted to specific Gram negative bacteria (Koval and Hynes, 1991; Hobley et al., 2006; Sockett, 2009). It is capable of killing many antibiotic-resistant, clinical pathogens including *Acinetobacter baumannii* and *Klebsiella pneumoniae in vitro*, and reduces the general bacterial burden *in vivo* (Negus et al., 2017). This bacterium has been studied by a limited number of scientists since the 1960s but has, with the rise of antimicrobial resistance (AMR), gained increasing attention. *B. bacteriovorus* is now being extensively investigated for its predatory and proteolytic properties, as well as its potential as a live antibiotic (Sockett and Lambert, 2004; Baker et al., 2017; Negus et al., 2017; Shatzkes et al., 2017). Previous studies have also shown positive correlations between the presence of *B. bacteriovorus* and health (Iebba et al., 2013), suggesting it may act as an environmental balancer, aiding in sustaining a beneficial microflora and thus contributing to good health (Pérez et al., 2016).

We recently described the novel serine protease BspK (*Bdellovibrio* serine protease K), from *B. bacteriovorus*, having kgp-like qualities (Vincents et al., 2011) hydrolyzing peptides and proteins after lysines (e.g., K). Not only did the enzyme act upon all lysine bonds in proteins during denaturing conditions, but specifically hydrolyzed IgG_1_ in the hinge region during native conditions (Bratanis et al., 2017). Our study highlighted the applicability of bacterial enzymes as biotechnological tools for quality control of biological therapeutic agents (biologics), such as monoclonal antibodies (mAbs). Pharma commonly apply mass spectrometry (MS) to analyze and quality control mAb-products, a method enhanced by proteolytic digestion of the sample to smaller peptides prior to analysis. These digestions are usually performed with various bacterial enzymes including trypsin and IdeS (Gupta et al., 2010; An et al., 2014; Zhang et al., 2016). During our previous work, we identified not only activity against human IgG, in the lysate from *B. bacteriovorus*, but also specifically against IgA. There are several bacteria known to produce IgA-specific proteolytic enzymes, including *Neisseria meningitidis, Haemophilus influenzae, Streptococcus pneumoniae*, and *Streptococcus sanguis* (Kornfeld and Plaut, 1981; Woof and Russell, 2011). Nevertheless, the enzymes produced by these bacteria are highly specific for the IgA hinge region, whereas *B. bacteriovorus* seemed to target the C-terminal end of the pIgA Fc-region.

Immunoglobulin A (IgA) is most commonly recognized as an anti-inflammatory antibody, predominantly found at mucosal membranes and in secretions, mediating protection against invading microorganisms by neutralization, agglutination and clearing. More recently it has become evident that IgA also induces effector functions such as phagocytosis, antibody-dependent cell-mediated cytotoxicity (ADCC) and release of inflammatory mediators, through interactions with Fc alpha receptor I (FcαRI) (Otten and van Egmond, 2004; Bakema and van Egmond, 2011; Woof and Russell, 2011). FcαRI, one of many IgA receptors, is expressed on cells of the myeloid lineage including neutrophils, monocytes, certain macrophages and dendritic cells, as well as platelets (Monteiro and Van De Winkel, 2003; Otten and van Egmond, 2004). Ligand binding to FcαRI is induced by inside-out signaling and has been shown to be increased after stimulation with cytokines such as IL-4 and IL-5. Importantly, IgA immune complexes (ICs) or clustering of the antibody also increases the affinity and avidity of the antibody to FcαRI (Otten and van Egmond, 2004; Bakema and van Egmond, 2011).

In circulation, IgA is referred to as plasma or serum IgA (pIgA). pIgA is primarily monomeric and exists as two main subclasses, IgA1 and IgA2, and two additional allotypes IgA2m(1) and IgA2m(2). IgA shares the common immunoglobulin (Ig) structure, arranged into two identical antigen binding Fab-regions linked to the effector mediating Fc-region through the flexible hinge region. The general Ig-architecture consists of two heavy chain-light-chain heterodimers, with the common immunoglobulin-fold secondary structure. Unlike IgG however, IgA in common with IgM has an additional 18 amino acid C-terminal tail on the heavy chain that is essential for polymerization. In turn, Secretory IgA (SIgA) is formed through the translocation of dimeric IgA (dIgA) across mucosal epithelial layers to external secretions (Royle et al., 2003; Woof and Russell, 2011).

Structural differences between the subclasses include an extended hinge region in IgA1 which is heavily O-glycosylated compared to IgA2, Both IgA subclasses carry N-glycans at position Asn^263^ (CH2) and Asn^459^(Fc-tail), however IgA2 carries an additional two or three glycosylation sites, depending on the allotype, at position Asn^166^ (CH1), Asn^337^ (CH2) and Asn^211^ (CH2) (Mattu et al., 1998; Bakema and van Egmond, 2011; Woof and Russell, 2011). The overall IgA N-glycosylation is, as described in the literature, predominantly represented by digalactosylated biantennary complex type glycans. A large portion (∼64%) of the terminal galactose residues are also sialylated, in contrast to IgG where only a minor fraction of the antibodies are sialylated (5–10% mono-, and 1% di-sialylated) (Mattu et al., 1998; Woof and Russell, 2011; Le et al., 2016).

The role of antibody glycosylation has been extensively studied for IgG, and it is well-known that removal of, or modifications to the composition of the IgG glycan at Asn^297^ of the CH2 domain affects receptor interactions and thus modulates downstream effector functions (Vidarsson et al., 2014; Lu et al., 2015; Li et al., 2017). By contrast, this likely does not translate to IgA, as the glycosylation is not critical for interactions between IgA and FcαRI (Mattu et al., 1998; Bakema and van Egmond, 2011).

The use of bacterial enzymes as biotechnological tools is already well-established and their application is wide-spread within both basic science and various industries. Two well-known examples are the cysteine proteinase IdeS (IgG-degrading enzyme of *Streptococcus pyogenes*) which specifically cleaves IgG in the hinge region, and EndoS (Endoglycosidase in *Streptococcus pyogenes*) which specifically hydrolyzes the conserved N-linked glycan of IgG impairing IgG mediated effector functions (Collin and Olsén, 2001; von Pawel-Rammingen et al., 2002; Johansson et al., 2008; Yang et al., 2010). We propose that *B. bacteriovorus*, with its large array of hydrolytic proteins, can act as an excellent source for further identification of more novel biotechnological tools. In this paper, we have followed up on our earlier findings suggesting that *Bdellovibrio* expresses an IgA hydrolyzing enzyme, identified the IgA-protease, characterized it on a molecular level, and taken advantage of its specificity to study pIgA Fc-tail glycosylation.

## Materials and Methods

### Bacterial Strains and Growth Condition

*Bdellovibrio bacteriovorus* HD100 was propagated and routinely cultured with *E. coli* TOP10 as prey, as previously described by Lambert and Sockett (2008). Lysis was determined by visual clearing of the culture medium as compared to a non-infected *E. coli* control. *E. coli* strains TOP10 and BL21(DE3) pLysE were used for the cloning and expression of recombinant proteins, respectively. *E. coli* was cultured in LB medium supplemented with 100 μg/ml ampicillin and 34 μg/ml chloramphenicol where applicable.

### Partial Purification of BspE From *Bdellovibrio* Lysate

Sterile filtered (0.22 μm) *B. bacteriovorus: E. coli* (predator: prey) o/n lysate was precipitated by a two-step ammonium sulfate precipitation (40–60%). Precipitated proteins were re-solubilized in 20 mM sodium phosphate (NaP) buffer pH 7.4, followed by a two-step ultrafiltration (Amicon Ultracel-100 flow-through and Ultracel-30 concentrate) (Merck Millipore, Ltd., Tullagreen, Ireland). Proteolytic activity and purity were verified by SDS-PAGE.

### Enrichment of BspE From Partially Purified *Bdellovibrio* Lysate

The partially purified *Bdellovibrio* lysate was pre-incubated with aprotinin (Thermo Scientific, Rockford, IL, United States), after which ActivX TAMRA-FP Serine Hydrolase Probe (Thermo Scientific) was added to the reaction according to manufacturer’s instruction. The sample was separated by electrophoresis, protein bands were detected using fluorescence detection in a ChemiDoc MP Imager (Bio-Rad, United States) (ChemiDoc) and identified by mass-spectrometry.

### In-Gel Digestion and Mass Spectrometry

Bands of interest (e.g., fluorescent bands), corresponding to the predicted size of BspE were excised from the acrylamide gel. The gel was sectioned into smaller pieces followed by de-staining, washing, and dehydration with acetonitrile (ACN, 50%). Reduction of disulfide bonds with TCEP solution (10 mM in 100 mM ammonium bicarbonate pH 8.0) and alkylation of cysteines with iodoacetamide (IAA, 15 mM in 100 mM ammonium bicarbonate pH 8.0) was followed by trypsin (Promega) digestion. Finally, peptides were extracted from the acrylamide matrix with formic acid (FA) and ACN (50%). Peptides were analyzed by LC–MS/MS and screened against the proteome of *B. bacteriovorus* HD100.

### Recombinant Expression and Purification of BspE

Codon optimized (*E. coli*) gene constructs representing BspE lacking a signal peptide were inserted into the pMAL-c5X plasmid (pMAL-c5X-*bd2692-His*) (GenScript, United States). BspE was recombinantly expressed in *E. coli* BL21(DE3) pLysE cells as a fusion protein with an N-terminal MBP-tag and a C-terminal His-tag. Bacteria were cultured (37°C, 225 rpm) until OD_620_ reached 0.4 when recombinant protein expression was induced with 0.2 mM IPTG. Protein expression was continued for 3 h (20°C, 225 rpm), after which the cells were collected and frozen. Frozen cells were thawed and resolved in His binding buffer (20 mM NaP pH 7.4, 500 mM NaCl, 20 mM imidazole), and sonicated for release of intracellular proteins (5 min × 5 min with an equally long pause between the sonication steps, 70% efficiency, kept on ice). Cell debris was removed by centrifugation. Sterile filtered supernatant was affinity purified on His GraviTrap columns (Fisher Scientific) and re-buffered to 20 mM Tris-HCl pH 8.0 on a PD-25 column (Fisher Scientific). The concentration of the proteins was determined using the Nanodrop (NanoDrop, Spectrophptometer-ND1000) and purity estimated through SDS-PAGE.

### Biochemical Characteristics of BspE

The optimal biochemical conditions for BspE enzymatic activity was evaluated. The effect of temperature (4–55°C), incubation time (0 min–24 h), NaCl (50–1000 mM), ions (0–10 mM CaCl_2_, MgCl_2_, MnCl_2_, and ZnCl_2_) and detergents [SDS, Tween-20, and Triton X-100 (0.1–1%); and Urea (1–4 M) and DTT (1–10 mM)] on BspE activity was determined using a fluorescence-based assay with a casein derivative substrate (Molecular Probes’ EnzCheck Protease Assay Kit, Thermo Fisher Scientific) or SDS-PAGE analyzing BspE activity on pIgA with densitometric quantification using Image Lab software (Version 5.2.1, Bio-Rad laboratories). pH-dependence was addressed by using sodium acetate buffer pH 5.0–5.5 and Tris buffer pH 6.0–9.0 followed by densitometric quantification. Characterization of the proteolytic activity was investigated with a panel of protease inibitors: AEBSF, ALLN, antipain, bestatin, chymostatin, E64, EDTA-Na_2_, leupeptin, pepstatin, Phosphoramidon, and PMSF (G-Biosciences, Geno Technology, Inc., United States) according to the manufacturer’s instruction followed by analysis using the above described fluorescence-based assay. Fluorescence was read (485 nm/535 nm, 0.1 s) using a microplate reader (VICTOR3 1420 Multilabel counter, Perkin-Elmer, United States).

### Autoproteolytic Activity of Recombinant BspE

BspE was incubated at RT for time-points ranging from 0 to 24 h, followed by analysis with SDS-PAGE under reducing conditions. Proteins were transferred to PVDF-membranes using the Trans-Blot Turbo equipment (Bio-Rad Laboratories). Membranes were blocked with 5% (w/v) blotting-grade blocker (Bio-Rad Laboratories) in PBST. Antibodies directed to MBP (Anti-MBP monoclonal, mouse antibody HRP-conjugated, NEB) or the His-tag (Anti-6x His-tag, mouse antibody, Abcam) (and Alexa Fluor-568 conjugated Donkey anti-Mouse IgG Antibody, Thermo Scientific) were used to visualize the fusion proteins in a ChemiDoc MP Imager (Bio-Rad, United States). Further analysis was performed by casein zymography. The samples were analyzed on a casein gel under non-reducing conditions. The gel was renatured (2.5%; Triton X-100) and subsequently incubated at 37°C, o/n, with developing buffer (50 mM Tris-HCl pH 7.5, 200 mM NaCl, 5 mM CaCl_2_, 1 μM ZnCl_2_). The gel was stained (PageBlue Protein staining solution, Thermo Fischer Scientific) and destained using mQ H_2_O.

### Proteolytic Activity on Native Immunoglobulins and Abundant Plasma Proteins

BspE was incubated with the human immunoglobulins pIgA, SIgA, IgG (1–4), IgE, IgM and IgD, as well as the plasma proteins albumin and orosomucoid. Digestions were performed o/n at 37°C in PBS. Enzymatic activity was determined following analysis by SDS-PAGE under reducing conditions. Native pIgA purified from plasma (Calbiochem, United States); human myeloma IgG 1,2, 4 (kappa chain) and human myeloma IgG3 (lambda chain) (Sigma Aldrich); human myeloma IgE (BioRad); human serum IgM (Sigma Aldrich); natural human IgD (Abcam, Cambridge, United Kingdom) were used.

### Preferential Sites of Hydrolytic Activity

Screening with several peptides (H2686, glycodrosocin, MOG) and the protein Apo-myoglobulin was performed to identify any preferential sites for BspE hydrolytic activity. Peptides were incubated overnight with BspE in PBS at 37°C. Fragments were separated through LC (Agilent Technologies 1290 Infinity) (C18) and analyzed by MS/MS (Bruker Impact II). Enzymatic cleavage sites were mapped using the software Bruker Compass Data Analysis 4.4 and BioPharma Compass 2.0. Preference of amino acids was calculated based on number of cleavages divided by total sum of the area under the peak for said amino acids (e.g., the possible differences in ability to fly for the peptides could not be considered). The precise specificity may however be biased due to the use of peptides with a high prevalence of certain amino acids. Further, due to the inability to measure exact quantities of hydrolytic peptide fragments by using mass spectrometry (e.g., not being able to consider differences in ability to fly of the peptide fragments by lack of heavy peptides), the precise order of the measured specificity may be slightly affected.

### Determination of BspE Hydrolysis Site in Plasma IgA

Plasma IgA (10 μg) was incubated with PBS or BspE (1 μg) o/n at 37°C together with a Pro-Pro-Y-Pro endoproteinase (1 μg; MoBiTec, Göttingen, Germany). The substrate was reduced in the presence of DTT (15 mM) for 60 min at 37°C. The fragments were separated on a C4 column (Agilent) and desalted in-line prior to ESI Q-TOF on a Bruker Impact II MS. Data was analyzed by the Data Analysis Software v 4.4 (Bruker).

### Removal of N- Glycosylation at Position Asn^459^ Does Not Affect BspE Hydrolysis of IgA

N-Glycans were cleaved with PNGaseF under non-reducing conditions according to manufacturer’s instruction (New England Biolabs). Cleaved glycans and enzyme were removed by ultrafiltration (Amicon Ultracel-50), followed by incubation with BspE and analysis by SDS-PAGE. Proteins were transferred to PVDF-membranes using the Trans-Blot Turbo equipment (Bio-Rad Laboratories). Membranes were blocked with lectin buffer (10 mM HEPES supplemented with 150 mM NaCl, 1% Tween 20, 0.01 mM MnCl_2_, 0.1 mM CaCl_2_). Fluorescein labeled Lens Culinaris Agglutinin (LCA-FITC) was used to confirm deglycosylation.

### Site-Specific Characterization of pIgA N-Glycosylation at Position Asn^459^

Human pIgA was incubated with BspE in PBS at 37°C, o/n. The released Fc-tail fragment and glycans were separated from the remaining pIgA molecule by ultrafiltration (Amicon Ultracel-50). The flow-through was subsequently digested with PNGaseF for N-glycan release. IgG and pIgA control samples digested with PNGaseF were also included. Released N-glycans were separated by filtration (PALL Nanosep, 10K) and fluorescently labeled with 2-aminobenzamide (2-AB) for analysis by hydrophilic interaction chromatography (HILIC) (Agilent Infinity 1260 using the AdvanceBio Glycan Mapping, 2.1, 150 mm, 2.7 μm, LC column, Agilent, United States). Glycan structures were annotated following an exoglycosidase array using α(1, -2,-3,-4,-6)Fucosidase (Megazyme, United States), α(2-3,-6,-8,-9)neuraminidase (ABS) or combined α-neuraminidase (ABS) and β-galactosidase (1, -3, -4) (BTG) (ProZyme, United States). All enzymatic reactions were performed in appropriate buffers (according to manufacturer’s instruction) o/n at 37°C.

## Results

### IgA Proteases From *B. bacteriovorus* Can Be Identified by Protease Inhibitor Affinity Purification

**FIGURE 1:**
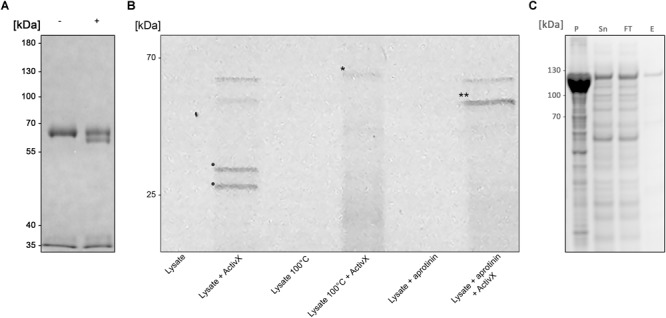
BspE from *Bdellovibrio bacteriovorus* **(A)** SDS-PAGE showing the enzymatic activity observed with crude *B. bacteriovorus* lysate on human plasma IgA (pIgA). Minus (–) and plus (+) signs denote absence and presence of crude *Bdellovibrio* lysate in the sample, respectively. **(B)** SDS-PAGE identifying Bd2962 from crude *B. bacteriovorus* lysate with ActivX Serine Hydrolase Probe. The addition of aprotinin resulted in inhibition of, and thus the elimination of two potential protease candidates (bands denoted with • in the second lane, as compared to the last lane). Further, the insensitivity to heating left only one band of interest for further characterization (the disregarded band is denoted with ^∗^ in lane four). The final band of interest is denoted in the figure with ^∗∗^. **(C)** Recombinant expression of BspE. The MBP-bd2906-His fusion protein is non-toxic and readily expressed in *E*. *coli.* The protein is partly found as inclusion bodies in the pellet (P) after cell lysis. Purification of the fusion protein from the supernatant (Sn) by Ni^2+^ affinity column purification resulted in loss of some protein in the flow through (FT). The eluted purified protein (E) was of high purity > 95% (as determined by visual inspection).

An initial enzymatic activity was observed with crude *B. bacteriovorus* lysate on human plasma immunoglobulin A (pIgA), suggesting a cleavage in the constant Fc domain (Figure 1A). Further analysis of the activity on pIgA confirmed hydrolysis in the CH3 domain resulting in a ∼5 kDa shift of the pIgA heavy chain, visualized by SDS-PAGE (under reducing conditions).

The lysate was fractioned based on biochemical properties, including solubility (ammonium sulfate precipitation), charge (ion-exchange chromatography), size (ultrafiltration), and protease inhibitor sensitivity, followed by analysis with SDS-PAGE under reducing conditions. Enzymatic activity was inhibited by ALLN, AEBSF, antipain, chymostatin, and PMSF; well-known inhibitors of cysteine (ALLN) and serine proteases (all others). The activity was however not blocked by addition of the serine protease inhibitor aprotinin (data not shown).

To identify the enzyme of interest we affinity purified the protein based on its property as a serine protease, exploiting its insensitivity to aprotinin. Pre-incubation of the crude lysate with aprotinin (Thermo Scientific, Rockford, IL, United States) to block general serine proteases, followed by incubation with the fluorescent serine hydrolase probe ActivX TAMRA-FP, allowed for selective labeling of aprotinin-insensitive serine proteases (Figure 1B).

The mass spectrometry analysis of labeled proteins identified the protease of interest as the 52.98 kDa *B. bacteriovorus* protein Bd2692, earlier annotated as a protease with serine-type endopeptidase activity, inferred by homology (UniProt). BspE (Bdellovibrio elastase-like serine protease) is classified as a peptidase S8 subtilisin like protein, containing a conserved peptidase S8 domain ranging from S_123_-T_365_ (E-value 1.65e-110) (NCBI GenBank), as well as a signal peptide with a predicted N-terminal cleavage site between amino acid 18–19 (Bendtsen et al., 2004). There are no additional conserved domains in the protein sequence. The catalytic triade, in the active site of BspE, has been predicted to be the Serine333-Histidine130-Aspartate132 motif^1^.

### A Partly Soluble and Active BspE Can Be Recombinantly Expressed in *E. coli*

Despite being annotated as a subtilisin, BspE was seemingly non-toxic to *E. coli* during recombinant expression of an MBP/His-fusion protein with Bd2692 devoid of its signal peptide. After affinity purification on a His GraviTrap column the fusion protein displayed a high purity >95% (as determined by visual inspection of SDS-PAGE) (Figure 1C). Autoproteolytic fragments of BspE were also observed in the purified material. A considerable amount of the material was found as inclusion bodies after cell lysis, and even more of the protein was lost in the flow through during affinity purification. One liter *E. coli* generated approximately 0.5 mg purified soluble protease.

### BspE Displays Autoproteolytic Activity

**FIGURE 2:**
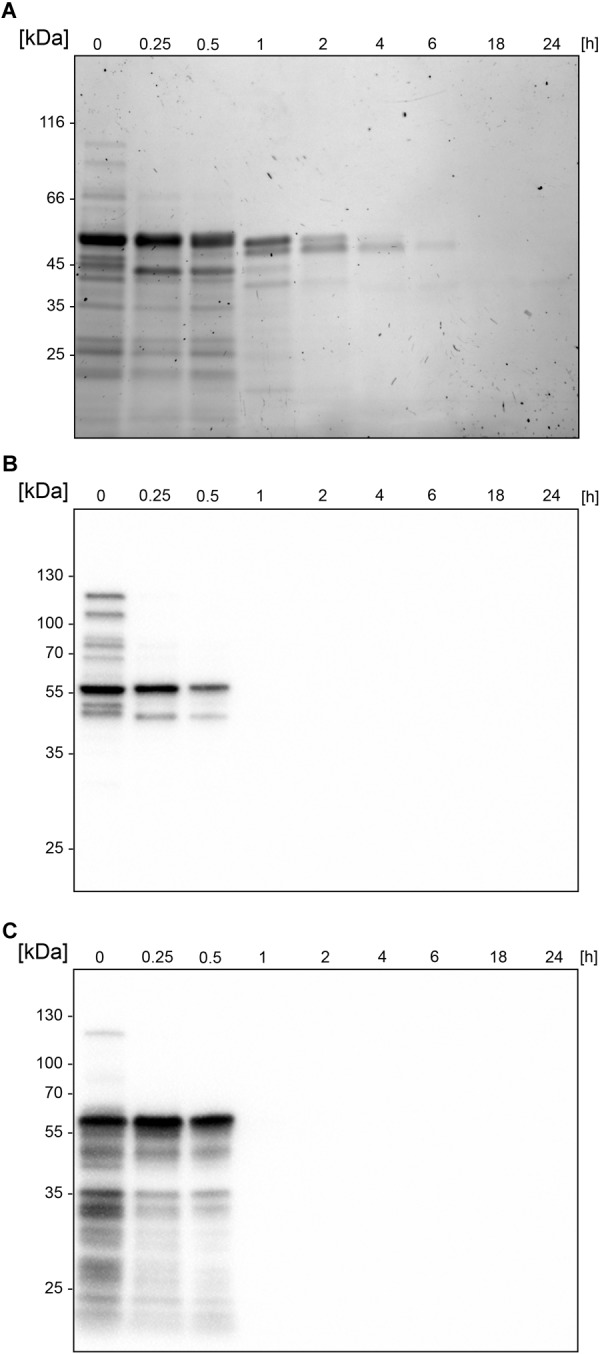
Autoproteolytic activity leads to an immediate fragmentation of BspE. SDS-PAGE and Western blots showing the autoproteolysis of the MBP-BspE-His fusion protein. **(A)** SDS-PAGE, PAGE-Blue staining showing continuous degradation of the protein (already in starting material). **(B)** Anti-MBP Western blot showing the gradual loss of MBP-signal over time. **(C)** Anti-His Western blot showing an immediate loss of the His-signal. The molecular weight of the intact recombinant BspE ∼96 kDa; MBP has a molecular weight of 42.5 kDa.

**FIGURE 3:**
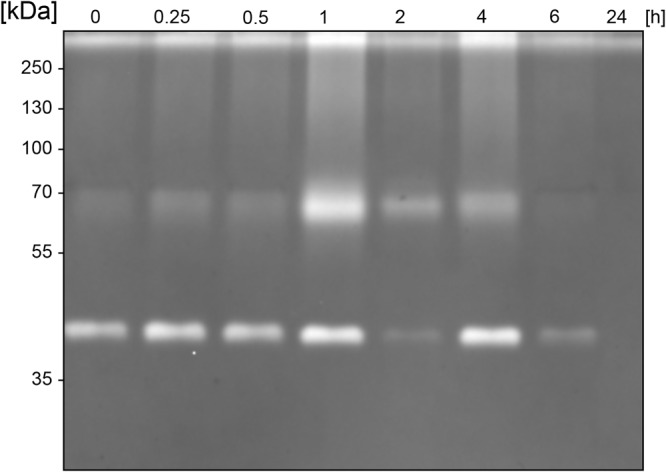
Sustained activity of BspE autoproteolytic fragments. Casein zymography showing the sustained activity of two BspE fragments with molecular weights of ∼70 and ∼40 kDa following autoproteolysis. The zymography shows a diminishing activity up to 6 h.

Similarly, to most general proteases, BspE displays autoproteolytic activity and self-degrades until it is undetectable with Western blot (against MBP- or His-tag). The autoproteolysis starts already during the protein expression and purification process, as seen on the SDS-PAGE (Figure 2A) and the Western blots (Figure 2B,C) where almost no full-length fusion protein can be detected (95.7 kDa). A vast majority of the full-length protein is however found as inclusion bodies in the pellet fraction (data not shown). The results show complete hydrolysis from the N-terminal end already at 1 h, seen by the detection of MBP (42 kDa) alone (Figure 2B). The rapid loss of the His- signal indicates that BspE is also trimmed C-terminally (Figure 2C). The casein zymogram confirms the activity of the autoproteolytic BspE fragments with molecular weights of ∼70 and ∼40 kDa, and a sustained activity over time (Figure 3).

### BspE Activity on pIgA Is Highly Influenced by Presence of Divalent Cations

**FIGURE 4:**
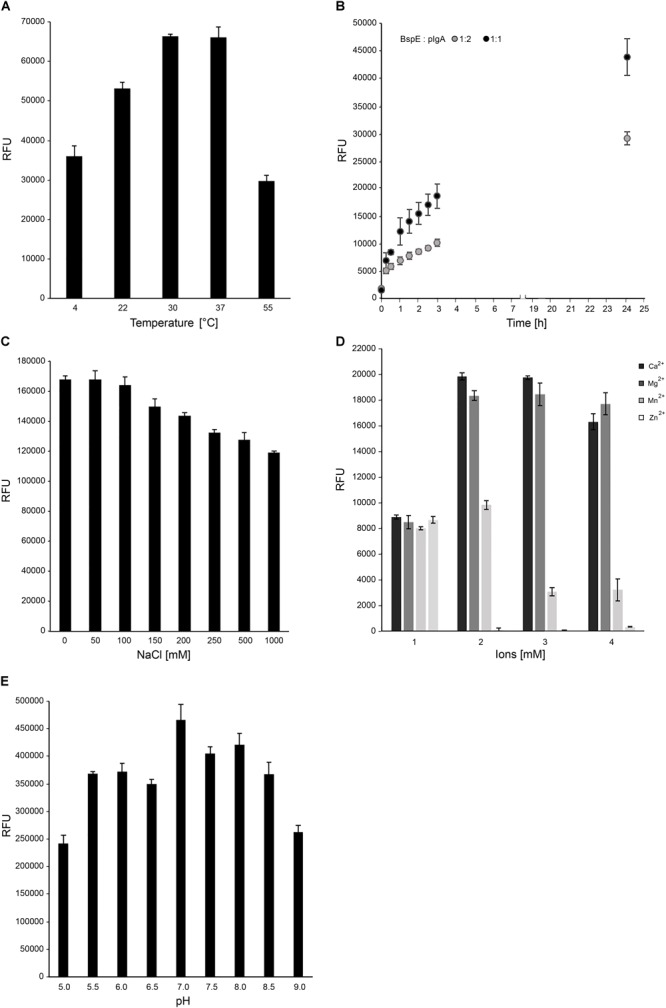
Optimal biochemical conditions for BspE activity. **(A)** BspE activity at increasing temperatures (4–55°C). **(B)** BspE activity over time at 37°C (0–24 h). **(C)** BspE activity with increasing concentrations of NaCl (0–1000 mM). **(D)** The effect of Ca^2+^, Mg^2+^, Mn^2+^, and Zn^2+^ at increasing concentrations (0, 2, 5, 10 mM) on BspE activity. **(E)** BspE activity at increasing pH, ranging from 5.0 to 9.0. Assays **(A–C)** were analyzed using the fluorescence-based assay EnzCheck Protease Assay. Assays **(D,E)** were analyzed by SDS-PAGE and densitometrically quantified. Enzymatic activity was measured as relative fluorescence units (RFU). Data is plotted as mean values ± SD.

Optimal biochemical conditions for BspE activity was investigated by using both fluorescence-based analysis and electrophoresis with densitometric quantification. The activity of BspE peaked at 30–37°C but was also present at both lower (4°C) and higher (55°C) temperatures (Figure 4A). Substrate hydrolysis could be detected already after 15 min with continued hydrolysis of the substrate with prolonged incubation (Figure 4B). Increasing concentrations of NaCl (50–1000 mM) did not reduce the enzymatic activity considerably (Figure 4C). The effects of divalent cations on the activity of BspE was investigated by an addition of CaCl_2_, MgCl_2_, MnCl_2_, and ZnCl_2_ (0–10 mM) to the reaction buffer. Addition of CaCl_2_ or MgCl_2_ increased the hydrolysis even at low molar levels, while MnCl_2_ and ZnCl_2_ partly or completely inhibited the enzymatic activity of BspE, respectively (Figure 4D). While having the highest activity at neutral pH BspE retained much of its hydrolytic capacity even at lower (5.0) and higher (9.0) pH (Figure 4E). The addition of detergents to the enzymatic reaction resulted in a general reduction or complete loss of BspE activity. The addition of SDS (0.1–1%), Tween-20 (0.1–1%), as well as the higher concentrations of DTT (10 mM) and urea (4 M) resulted in a general loss of activity on pIgA, and a loss of autoproteolytic activity to various degrees. The enzymatic activity was retained with the lower concentrations of DTT (1 mM), and slightly reduced by the presence of Triton X-100 (0.1–1%) and urea (1 M) (Supplementary Figure S1).

### pIgA Can Be Site-Specifically Hydrolyzed by BspE

**FIGURE 5:**
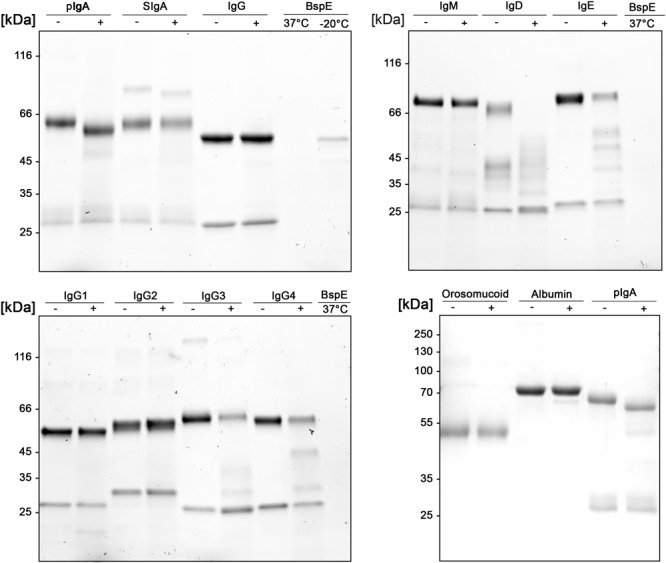
Native human immunoglobulins are not general BspE substrates. Native human immunoglobulins including: IgA, SIgA, IgE, IgD, IgM, and all the IgG subclasses were incubated with BspE (37°C, o/n), + and – denotes presence and absence of BspE. Analysis by SDS-PAGE under reducing conditions.

A more detailed investigation in regards to the substrate specificity of BspE was initiated by incubating all the human immunoglobulin classes (IgG, pIgA, SIgA, IgE, IgM, and IgD), the IgG subclasses (1–4), and the plasma proteins albumin and orosomucoid with the enzyme (Figure 5). The results showed an enzymatic activity toward the C-terminal end of the native pIgA Fc-region, releasing the Fc-tail with its glycan at position Asn-459, seen as a clear shift of the antibody heavy chain (Figure 5). Single-site cleavage was also observed toward the secretory component (SC) of SIgA, and albumin. Proteolysis of other native immunoglobulins and plasma proteins was either absent (IgG1 and IgG2, IgM and orosomucoid) or unspecific with multiple cleavage sites (IgG3 and IgG4, IgD and IgE).

### BspE Is a Broad-Spectrum Protease With a Low Level of Amino Acid Bond Specificity

**FIGURE 6:**
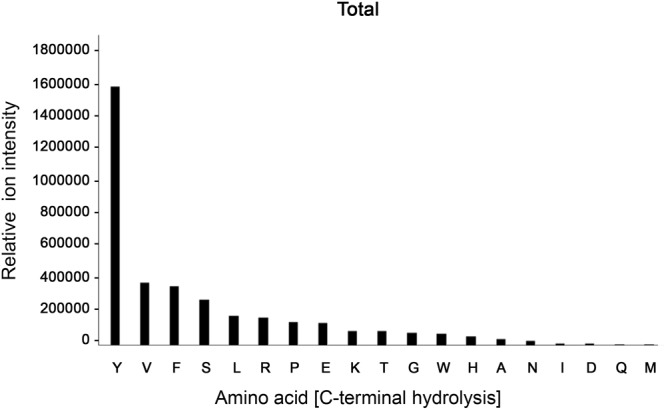
BspE displays broad activity on shorter peptides and denatured proteins. The combined results following screening of BspE activity on several peptides (MOG, and myoglobulin, H2686, and glycodrosocin). Enzymatic cleavage sites were mapped, and preference of amino acids calculated. The cleavage profile for the individual peptides can be found as (Supplementary Figure S2) and sequences as (Supplementary Table S1). The x-axis shows hydrolysis c-terminally of denoted amino acid. The y-axis denotes ion intensity measured.

Despite its ambiguous activity on native immunoglobulins, BspE had a broad activity against most amino acid bonds in shorter peptides and denatured proteins, with a slight preference for hydrolysis C-terminal of Y, V, F, S, L, R, P, E, and K (Figure 6, Supplementary Figure S2 and Supplementary Table S1). The precise specificity may however be biased due to the usage of peptides with a high prevalence of these amino acids. However, it is clear that BspE can cleave most peptide bonds in a protein.

### Removal of N- Glycosylation at Position Asn^459^ Does Not Affect BspE Hydrolysis of IgA

**FIGURE 7:**
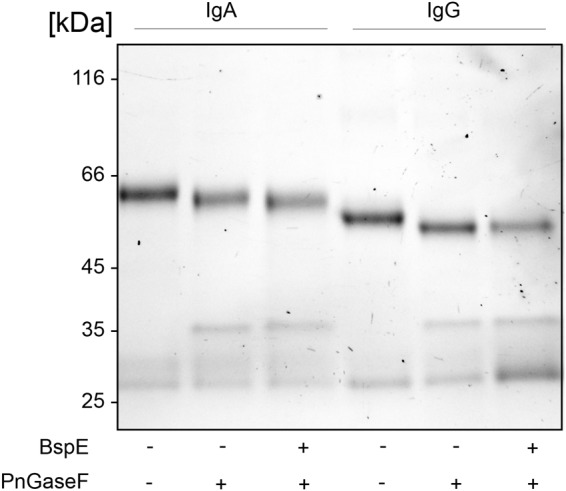
Removal of N- glycosylation at position Asn^459^ does not affect BspE hydrolysis of IgA. IgA control, IgA deglycosylated with PNGaseF (non-denaturing reaction conditions), IgA deglycosylated with PNGaseF followed by incubation with BspE were separated on a 10% SDS-PAGE. IgG verifies the activity of PNGaseF and the absence of BspE cleavage.

The proximity of the Fc-tail N-glycan to the determined BspE cleavage site on pIgA raised the question regarding the importance of the glycan for the enzymatic activity. Removal of the pIgA N-glycans prior to hydrolysis with BspE did not affect the enzymatic activity on pIgA. This indicates that the N-glycan at position Asn^459^ is not involved in the BspE hydrolysis of pIgA (Figure 7). Successful PNGaseF deglycosylation of IgA was verified by SDS-PAGE and LCA lectin blot prior to incubation with BspE (data not shown).

### BspE Hydrolyses Plasma IgA in the Fc-Tail

**FIGURE 8:**
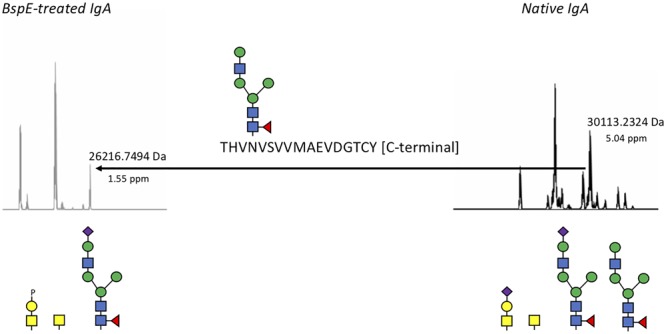
BspE hydrolyses plasma IgA in the Fc-tail. Native and BspE-treated plasma IgA were hydrolyzed with a PPTP-protease to separate the heavy chain in two fragments. The hydrolysis fragments were reduced and analyzed by mass spectrometry for intact masses. Generated ions were deconvoluted and the most prevalent ions compared to each other. The Fc-heavy chain fragment of native IgA had a mass of 30113.2324 Da corresponding to the full-length IgA1 heavy chain hydrolyzed at the PP/TP-site in the hinge. A loss of a mass corresponding to a P/T hydrolysis in the C-terminal part of the Fc fragment in combination with an N-glycan resulted in the 26216.7494 Da fragment generated after BspE hydrolysis. Putative glycan structures based on mass are depicted below the ion spectra. Mass differences between theoretic and observed masses are depicted below the observed masses as ppm values.

Likely due to the native conformation of pIgA, we only observed a single shift of pIgA after incubation with BspE. The cleavage site in pIgA, P/THVNVS, was mapped by LC–MS/MS and correlates well to the 5 kDa shift observed on SDS-PAGE following cleavage of pIgA with BspE (Figure 8).

### Site-Specific Characterization of pIgA N- Glycosylation at Position Asn^459^

**FIGURE 9:**
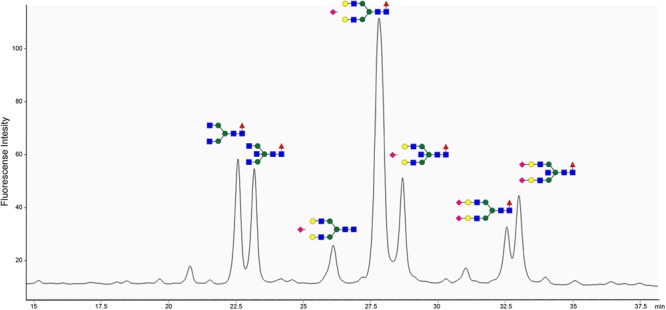
pIgA N-linked glycans are primarily represented by extended biantennary structures. HPLC of 2-AB labeled glycans of the pIgA Fc-tail cleaved by BspE, followed by glycan release with PNGaseF. Characterized N-glycan structures are annotated using the nomenclature of the Consortium for Functional Glycomics (blue square: *N*-Acetylglucosamine, green circle: mannose, yellow circle: galactose, purple diamond: sialic acid, red triangle: fucose). Site-specific characterization of 2-AB labeled pIgA N- glycosylation at position Asn^459^ by High Pressure Liquid Chromatography (HPLC). The y-axis indicates fluorescent intensity and the x-axis shows the retention time the individual glycoforms.

The site of BspE hydrolysis on pIgA also facilitated the separation of the pIgA Fc-tail containing the N-glycan attached to Asn^459^ from pIgA and facilitated site-specific characterization of the Asn^459^ N-glycan (Figure 9). The exoglycosidase array further enabled annotation of the specific N-glycan structures on the pIgA Fc-tail (data not shown) and revealed an abundance of extended, bi-antennary glycoforms, primarily represented by digalactosylated and mono- or di-sialylated glycan structures. The N-glycans were predominantly core-fucosylated, and to a varying extent also modified with a core GlcNAc (*N*-Acetylglucosamine).

## Discussion

The predatory bacterium *B. bacteriovorus* is the best studied predatory bacterium to date. In addition to its potential as a live antibiotic *B. bacteriovorus* produces a plethora of hydrolytic proteins, making it a potential source for novel antimicrobial and biotechnological agents. During our previous work on *B. bacteriovorus* we identified BspK, a serine protease with activity against human IgG (Bratanis et al., 2017), we also observed a specific cleavage pattern on human plasma IgA. Here we have identified and characterized the enzyme behind this phenomenon: BspE, a broad-spectrum serine protease, which despite its broad activity displays a specificity toward cleaving the Fc-tail on native pIgA.

Our results display BspE as an elastase-like, in general, broadly acting protease with activity on a wide range of denatured protein substrates. BspE lacks a distinctive and consistent cleavage pattern and the site of hydrolysis is dependent on the substrate. BspE has previously been identified in Host-independent *B. bacteriovorus* (HIB) supernatant, suggesting that this protease is not directly involved in or essential for *B. bacteriovorus* virulence or invasion of prey (Monnappa et al., 2014). However, it cannot be excluded that BspE is involved in hydrolysis of prey-derived substrates, by itself or in combination with other *B. bacteriovorus* enzymes. Its broad-spectrum hydrolysis could be favorable in the degradation of various prey proteins to enable nutrient uptake by the host and supplement the plethora of other secreted proteases by *B. bacteriovorus*. Alternatively, as *Bdellovibrio* produces a vast array of proteases, the broad activity of BspE could possibly also be involved in the degradation of other proteases, thus acting protectively against excessive proteolytic damage to *Bdellovibrio* self or the prey host. Though highly interesting, the biological function of BspE is however not the scope of this article. Nevertheless, our data in combination with a biological understanding suggests that pIgA is not the main substrate of BspE. We base this conclusion on several considerations including the concept of compartmentalization and factual results presented in this paper. *Bdellovibrio* is, besides aquatic and terrestrial environments, found in the gastrointestinal tract of mammalians, environments where this bacterium is unlikely to encounter pIgA (rather dIgA), as this antibody is mainly found in the circulation. However, the Ig-fold comprising the structure of immunoglobulins is a widely distributed protein conformation identified in both eukaryotes and prokaryotes, including *E. coli* and other enterobacteria known to colonize the gastrointestinal tract of mammalians (Schwudke et al., 2001; Iebba et al., 2013). Thus, *Bdellovibrio* is certain to encounter proteins with Ig-like folds. By this reasoning we suggest that the activity on pIgA most likely is coincidental, based on a resemblance with the true BspE substrate(s). The incomplete hydrolysis of pIgA also suggests that BspE requires a specific amino acid sequence and conformation of the pIgA Fc-tail in order to cleave the substrate. We speculate that a nick created by the proline in the secondary amino acid structure in pIgA gives the protease access at that specific site.

The role of IgG in the immune system has been extensively investigated and the importance of the conserved N-linked glycan at position Asn^297^ in the CH2 domain, for IgG effector functions, is by now widely accepted. It has also been shown that certain glycoforms can regulate the elicited immune response, e.g., antibodies enriched for terminal sialic acid residues have shown to have an increased anti-inflammatory activity, and removal of the core fucose residue increases antibody-dependent cell-mediated cytotoxicity (ADCC). These insights are now being applied to design therapeutic antibodies with increased efficacies (Golay et al., 2013; Mimura et al., 2018). Although much has been elucidated regarding the structural and functional role of IgG Fc-glycosylation, this knowledge does not directly translate to the other immunoglobulins. The general characteristics of Ig N- and O-glycosylation has by now been described for all the human immunoglobulins. However, knowledge about the specific biological importance of Fc-glycosylation for immunoglobulins other than IgG, is scarce. The extensive glycosylation of IgA has been comprehensively mapped, however there is little known about any functional roles of IgA glycosylation in regards to immunological responses (Hong et al., 2015). Here we propose the potential of BspE as a tool to specifically investigate the importance, and function of the C-terminal tail including its glycan for biological activities involving pIgA. The specific cleavage of the C-terminal part of the pIgA Fc-region, compared to other immunoglobulins cleaved by BspE, naturally raised the question if the carbohydrate structure at position Asn^459^ was involved in the enzymatic reaction. Our results showed that the removal of all the N-glycans on pIgA previous to treatment with BspE had no effect on the hydrolysis of the antibody. This suggests that the proximity of the N-glycan to the cleavage site is irrelevant for BspE cleavage of pIgA (Figure 7). Characterization of the Fc-tail N-glycan (Asn^459^) correlated well to what has been previously described for pIgA, with an abundance of extended, bi-antennary glycoforms, primarily represented by digalactosylated and mono- or di-sialylated glycan structures (Mattu et al., 1998).

The predicted size, based on amino acid composition, of the full-length MBP-BspE-His fusion protein was 95.7 kDa. Notably the affinity purified recombinant fusion protein was observed as somewhat larger when analyzed by SDS-PAGE (Figure 1C). Such discrepancies between predicted and observed migration of proteins on SDS-PAGE is not an uncommon phenomenon, and it is termed “gel shifting.” The underlying reasons are yet to be conclusively explained however inherent properties of the protein such as a high content of acidic amino acids, as well as properties affecting SDS-binding to the protein including high hydrophobicity and protein structure, have been proposed as potential explanations affecting the protein migration (Rath et al., 2009; Guan et al., 2015). As shown in the anti-MBP western blot (Figure 2B) the barely detectable intact fusion protein (MBP-BspE-His) at 0 h suggests that the autoproteolytic activity starts already during the expression and purification process of BspE. Additionally, the rapid loss of signal, and the presence of autoproteolytic fragments in the SDS-PAGE and Western blots (Figure 2) indicates a trimming from both the N- and C-terminal end of the protein. We propose BspE to be a zymogen as the trimming of the protein produces enzymatically active intermediate products, as shown by a sustained enzymatic activity over time (Figure 3, 4B). The removal of both the MBP- and His- tag by autoproteolysis is also a likely contribution to the poor affinity purification of BspE. Nevertheless, although the tags are rapidly removed they still contribute to the solubility and initial purification of the protein. The autoproteolytic property of BspE rendered it impossible to determine a definite (full length) BspE concentration in the performed activity assays. Based on densitometric estimations, an enzyme:substrate ratio of ca 1:100 was used for all fluorescence-based assays, and ca 1:10 for all densitometrically quantified assays. To minimize any discrepancies between experiments the purified protein was immediately aliquoted and stored at -20°C following purification, repeated freeze/ thaw cycles were avoided and the assays were consistently performed with the same batch of enzyme.

In this work we showed that the addition of Zn^2+^ and Mn^2+^ inhibited the enzymatic activity of BspE (Figure 4D). Histidine is known to bind both Zn and Mn, and it could be argued that the presence of the His-tag in the recombinant BspE fusion protein, could limit the inhibitory effect of the ions. Our results clearly show that Zn^2+^ completely inhibits the BspE activity already at 2 mM, while Mn^2+^ reduces the activity by approximately 50% at this concentration. Importantly, the affinity of the His-tag for Mn^2+^ is low compared to Zn^2+^, Co^2+^, Ni^2+^ and other transition ions commonly used for immobilized metal-affinity chromatography (IMAC) using a His-tag (Bornhorst and Falke, 2000; Ching et al., 2016). Thus, the presence of the His-tag does not seem to have a substantial impact on the inhibitory effect of the ions, and removing the His-tag would most likely only increase the inhibitory effect of these ions slightly.

The addition of detergents to the reaction resulted in reduced hydrolysis of pIgA, as well as alterations in the autoproteolytic cleavage pattern observed on the SDS-PAGE (Supplementary Figure S1). Based on our results showing a broad BspE activity against most amino acid bonds in shorter peptides and denatured proteins (Figure 6 and Supplementary Figure S2), taken together with the change in autoproteolytic cleavage pattern, the results suggest that the addition of detergents inhibit the enzyme rather than effecting the substrate. This suggests that the enzymatic activity of BspE is dependent on the intact three-dimensional protein structure, rather than certain amino acid sequences/peptides. Enzymes with a broad substrate specificity are not uncommon in nature and like enzymes in general their activity is dependent on the surrounding conditions including, temperature, pH, presence or absence of specific ions at different concentrations, the presence of other catalysts, etc. The enzymatic specificity of most enzymes is better addressed as a preference for certain cleavage sites, determined by hydrolysis following preferential amino acid residues, peptide sequences or structures. Pseudolysin from *Pseudomonas aeruginosa*, is a well-studied enzyme with a preference for aromatic and/or large aliphatic amino acids at the P1’ position, and a distinct bias against acidic residues at the P2’ position (Yang et al., 2015). Other examples of well-studied enzymes with wide cleavage specificities include cathepsin G, neutrophil elastase and the metalloproteinases (MMP’s) 2 and 9 (Polanowska et al., 1998; Doucet and Overall, 2011). BspE falls into this category of broad-spectrum proteases though the preferential amino acid bonds seem to be less specific. Importantly, even proteases with multiple and virtually unspecific cleavage preferences can have use in biotechnological applications when used in defined conditions and with single substrates.

In this paper we have described the novel protease BspE from *B. bacteriovorus*, with a focus on the activity on plasma IgA (pIgA). By hydrolyzing pIgA BspE cleaves the Fc-tail including the N-linked glycan at position Asn^459^. Besides contributing to the general understanding of *B. bacteriovorus* biology, we also propose that BspE could be used as a potential tool to investigate the importance of and biological function of the pIgA C-terminal tail-fragment, including mechanisms such as IgA agglutination and clearing of antigens, interactions at mucosal surfaces or with receptors, including the FcαR. Finally, this identification and characterization of BspE further emphasizes the potential of *B. Bacteriovorus* as a source for novel hydrolytic proteins with potential applications within both basic research and the life science industry.

## Data Availability

All datasets generated for this study are included in the manuscript and/or the Supplementary Files.

## Author Contributions

EB and RL conceived the project and wrote the manuscript. Both authors contributed to the data collection, data analysis, interpretation, and read and approved the final manuscript.

## Conflict of Interest Statement

The authors declare that the research was conducted in the absence of any commercial or financial relationships that could be construed as a potential conflict of interest.

## Footnotes

1 prosite.expasy.org
