# Supplementary material for: A Novel Broad-Spectrum Elastase-Like Serine Protease From the Predatory Bacterium Bdellovibrio bacteriovorus Facilitates Elucidation of Site-Specific IgA Glycosylation Pattern
Source: Front Microbiol. 2019 May 3;10:971. doi: 10.3389/fmicb.2019.00971 (PMC6510308; doi:10.3389/fmicb.2019.00971)
Supplement: Supplementary file 1 [file Data_Sheet_1.pdf]

SUPPLEMENTAL MATERIAL

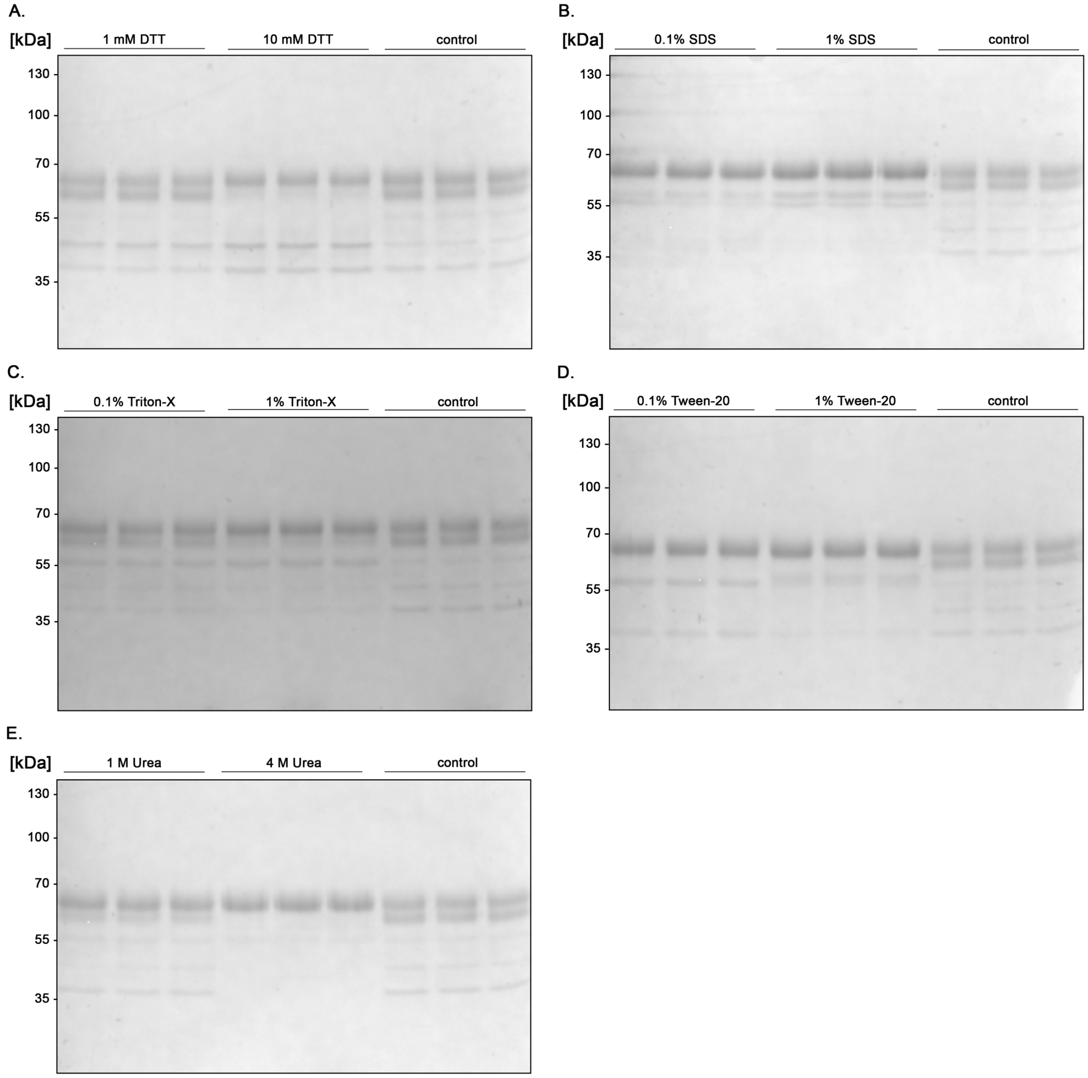

Fig. S1 Altered BspE cleavage patterns of IgA observed in the presence of detergents. **I**mpact of 1-10 mM DTT (A), B) 0.1-1% SDS (B), 0.1-1% Triton-X (C), 0.1-1% Tween-20, (D), or 1-4 M urea (E) on hydrolysis on IgA as elucidated by SDS-PAGE.

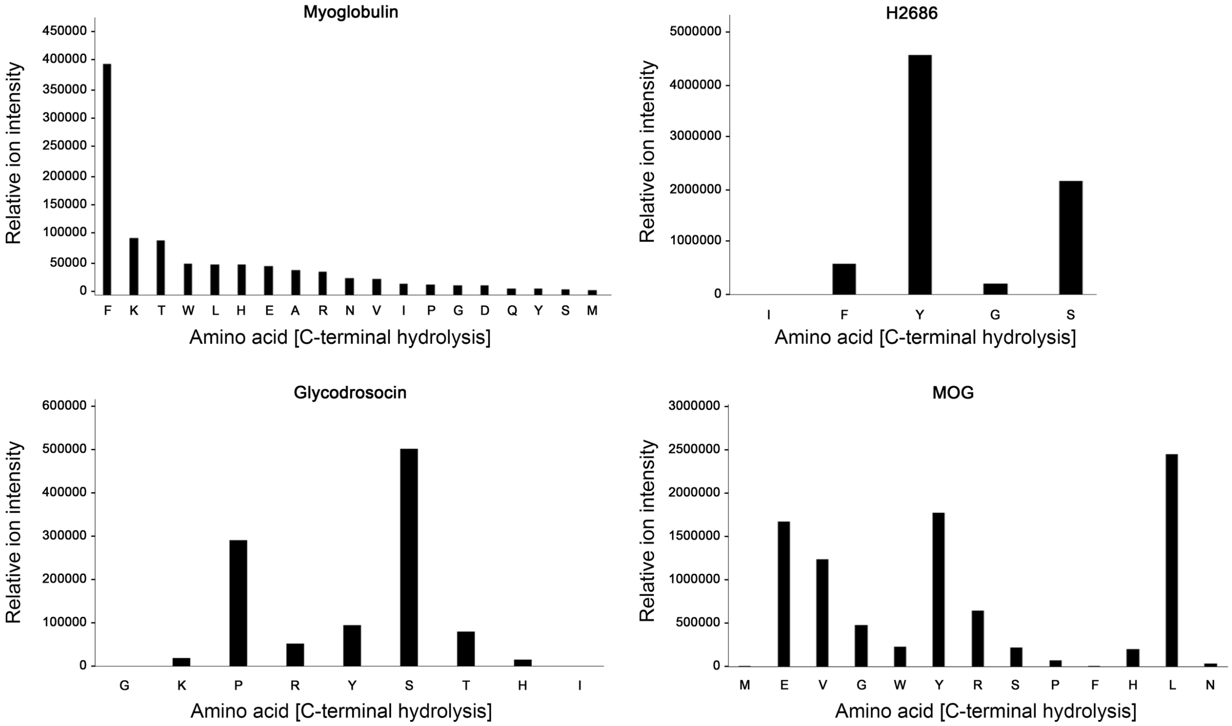

Fig. S2 Mapping preferential sites of hydrolytic activity. Enzymatic cleavage sites mapped on the individual peptides/ denatured proteins combined in Fig. 5. Enzymatic activity on myoglobulin (A), H2686 (B), Glycodrosocin (C) and MOG (D). Full sequence information of the peptides and proteins can be found in Table S1.

Table S1. Sequences of peptides and protein used to evaluate BspE preferential sites of hydrolysis. Screening through LC separation and MS/MS analysis with short peptides and Apo-myoglobulin to identify BspE preferential sites of hydrolytic activity. Lysines are marked in bold and the threonine in Glycodrosocin carrying a core 1 O-glycan is underlined.

**Peptide / protein**	**Sequence**
MOG	MEVGWYRSPFSRVVHLYRNG**K**
H2686	YIYGSF**K**
Glycodrosocin	G**K**PRPYSPRPTSHPRPIRV
Apo-myoglobulin	MGLSDGEWQQVLNVWG**K**VEADIAGHGQEVLIRLFTG<br>HPETLE**K**FD**K**F**K**HL**K**TEAEM**K**ASEDL**KK**HGTVVLTALG<br>GIL**KKK**GHHEAEL**K**PLAQSHAT**K**HKIPI**K**YLEFISDAIIHV<br>LHS**K**HPGDFGADAQGAMT**K**ALELFRNDIAA**K**Y**K**ELGF<br>QG
